# Why are all multi-centre trial sites not the same? A network analytic approach

**DOI:** 10.1186/1745-6215-16-S2-P195

**Published:** 2015-11-16

**Authors:** Helen McAneney

**Affiliations:** 1 Northern Ireland Network for Trials Methodology Research, Centre for Public Health, Queen's University Belfast, Belfast, UK

## Background

Why do some centres recruit, and/or retain, participants better than others? This is a question many trialists have pondered at one time or another. One aspect of enquiry, not often considered, is that of the interactions of the trial team themselves.

## Aim

To investigate whether social network aspects of members within in each centre, and how they interact as a team, have any bearing on recruitment and retention rates.

## Method

Assessment of a multi-centre trial, incorporating network data on communication and trust within the team, collected at discrete intervals, to gauge ‘team spirit’. Members within each centre will be asked to provide information on communication within the team network (e.g. email, face-to-face, etc.) regarding key aspects of support, organisation and resource sharing, both formal and informal.

Data will be assessed by actor oriented longitudinal network analysis techniques implemented in SIENA. This is a modelling framework to assess the outcome variable of recruitment and its association with ‘team spirit’, as measured through network parameters for communication and interaction, whilst controlling for known network structures such as reciprocity (i.e. if A interacts with B, then B interacts with A), hierarchical formal structures, and density. The same model will be applied to each centre and parameter estimates obtained. These will, in turn, form a basis for meta-analysis over all trial sites to factor in centre characteristics (e.g. size), and determine if higher level characteristics have importance.

Findings will provide key information for the planning and implementation of future trials.

